# Targeting H3K27me3 loss in pediatric brain tumors - a perspective on epigenetically guided cancer therapy

**DOI:** 10.18632/oncotarget.28427

**Published:** 2023-05-12

**Authors:** Michael Goldstein

**Keywords:** epigenetics, brain tumor, EZH2, H3K27me3, radiation therapy

High-grade tumors of the central nervous system including medulloblastoma, ependymoma and DMG (diffuse midline glioma, formerly known as DIPG (diffuse intrinsic pontine glioma)), constitute a major challenge in pediatric oncology [1–3]. They are characterized by an aggressive growth, high relapse rates and claim lives of many pediatric cancer patients. Both, medulloblastoma and ependymoma are treated with surgical resection followed by adjuvant radiation therapy [4, 5]. DMG, on the other hand, diffusely infiltrates the brain stem making a resection virtually impossible. Thus, radiotherapy is the primary treatment modality for this tumor [6].

Medulloblastoma has been divided into 4 molecular subgroups that differ with regard to their molecular profiles and treatment outcomes [7, 8]. Whereas WNT (Wingless/Integrated) medulloblastoma has the best prognosis with a 95% survival rate, SHH (Sonic Hedgehog) and group 4 tumors have intermediate prognosis with survival rates of 70% [2, 9–11]. In contrast, group 3 medulloblastoma has the worst outcomes with a survival rate of less than 50% [2, 9–11], which has been attributed to radiation and chemotherapy resistance of this subgroup resulting in frequent recurrences [2, 3]. Interestingly, posterior fossa ependymoma was also found to include multiple molecular groups that affect clinical outcomes [12, 13]. In this regard, Posterior Fossa B (PFB) tumors have an excellent prognosis while the PFA subgroup that constitutes over 70% of ependymomas has poor outcomes with a 10-year overall survival of less than 60% [14]. Unlike medulloblastoma and ependymoma, diffuse midline glioma is unresectable due to an infiltrative growth pattern within the brainstem. The only established treatment option for this brain tumor is radiotherapy [15]. While radiation temporarily attenuates the progression of DMG this brain cancer remains incurable and most children succumb to their disease [15].

**Figure 1:**
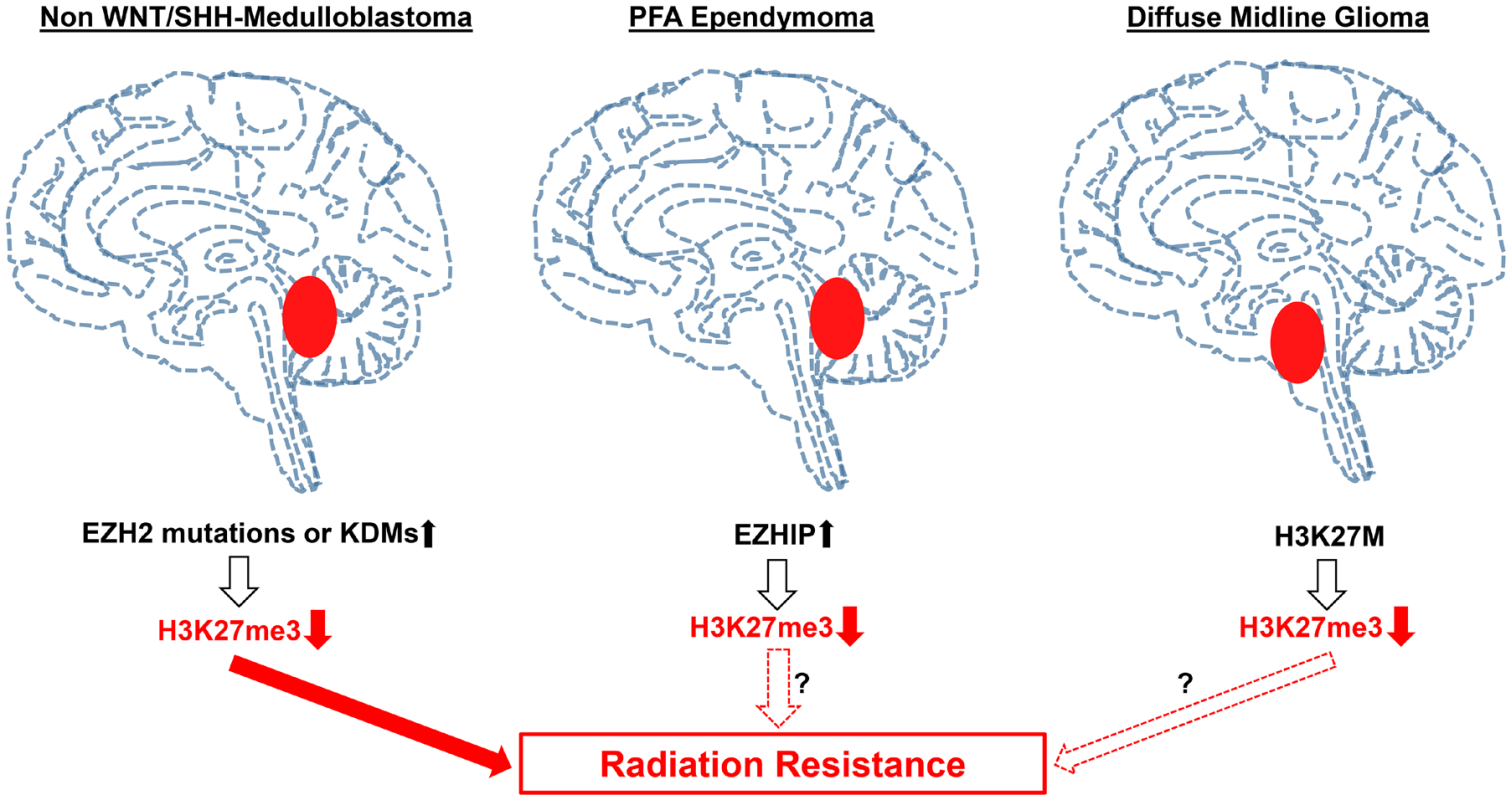
H3K27me3 loss in pediatric brain tumors. In non-WNT/SHH medulloblastoma EZH2 mutations and overexpression of H3K27me3-specific histone de-methylases (KDMs) lead to low H3K27me3 levels. In PFA ependymoma EZHIP overexpression inhibits EZH2 resulting in H3K27me3 loss. In DMG a dominant-negative H3K27M mutation exhibits an inhibitory effect on the PRC2 complex suppressing EZH2-dependent H3K27 tri-methylation.

The molecular profiles of the aforementioned pediatric brain tumors have been extensively investigated demonstrating distinct epigenetic traits (Figure 1). Strikingly, a global loss of H3K27 tri-methylation (H3K27me3) as a result of the dominant-negative histone H3K27M mutation was found to be a hallmark of DMG occurring in the majority of the tumors [15]. H3K27me3 is a product of the EZH2 histone methyl-transferase affecting multiple cellular processes including transcription, chromatin structure and DNA damage response [16]. Similarly, the aggressive PFA ependymoma subgroup is characterized by a lack of H3K27me3 due to an overexpression of the EZHIP protein that acts as an EZH2 inhibitor whereas less aggressive PFB tumors retain normal H3K27me3 levels [17]. However, no comprehensive analysis of H3K27me3 expression patterns in medulloblastoma has been performed and the significance of this epigenetic mark in pediatric brain tumors has remained unknown.

To address this, we have investigated the levels of the H3K27me3 histone mark and its role in treatment response of non-WNT/SHH medulloblastoma comprising group 3 and group 4 tumors [18]. We demonstrated that about 50% of the tumors in patients with group 3 and group 4 medulloblastoma are H3K27me3 deficient [18]. Strikingly, loss of H3K27me3 was associated with high relapse rates and a poor survival [18]. Using a model of group 3 medulloblastoma we show that H3K27me3 loss results in a profound radiation resistance consistent with the treatment resistant phenotype that we observed in H3K27me3-deficient medulloblastoma patients [18]. We went on to explore various cellular processes that can affect cancer cell response to DNA damage causing radiation resistance. We found that loss of H3K27me3 does not affect DNA repair, cell cycle progression or activation of cell death pathways. It led, however, to an excessive activation of the pro-survival AKT signaling pathway following DNA damage induction resulting in a radioresistant phenotype [18].

**Figure 2:**
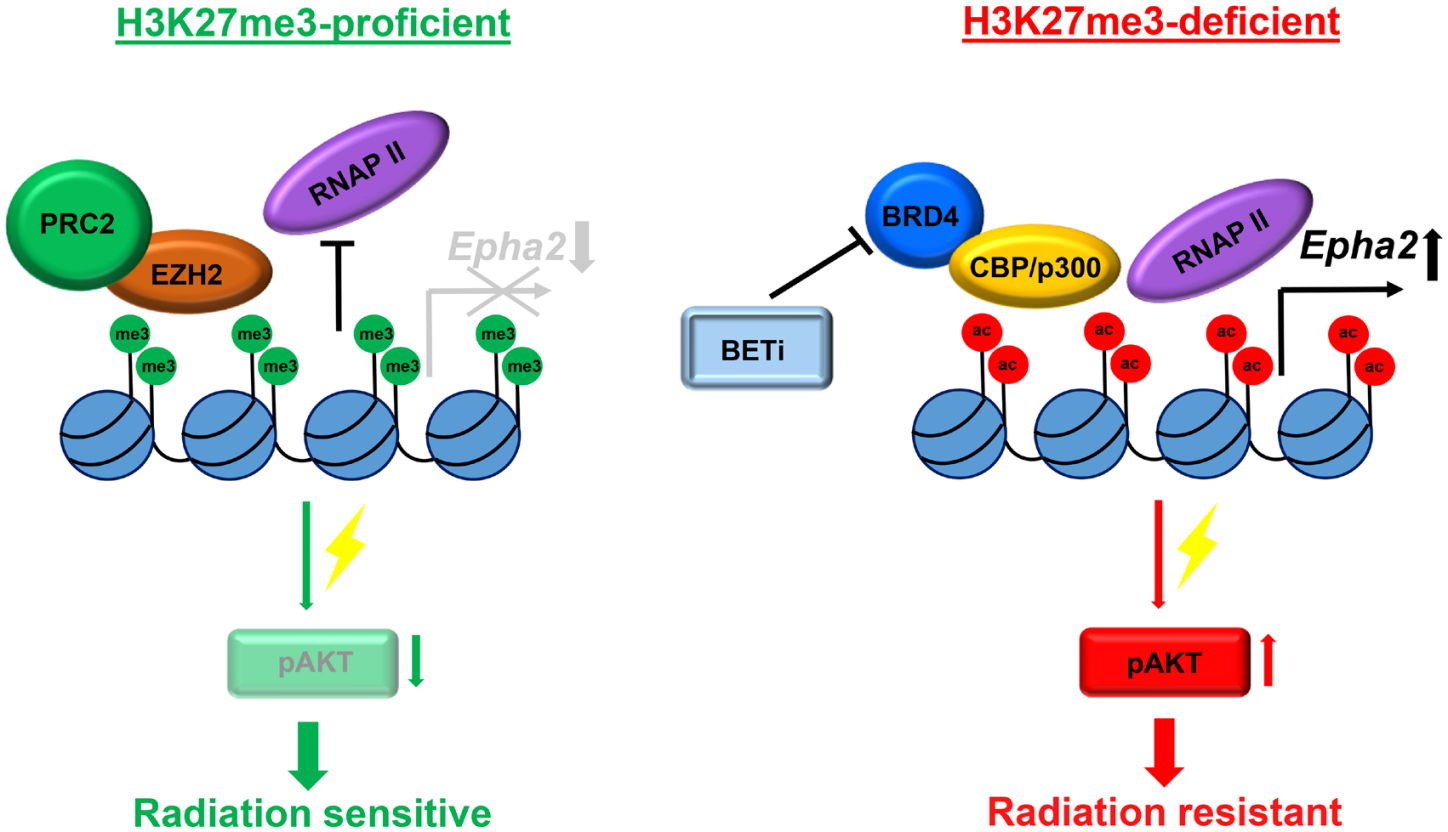
Mechanism of radiation resistance in H3K27me3-deficient medulloblastoma. In H3K27me3-proficient cells expression of the *Epha2* gene is blocked by high levels of transcriptionally repressive H3K27 tri-methylation within its promotor. DNA damage induced AKT signaling is attenuated resulting in cell sensitivity to radiation. In H3K27me3-deficient cells reciprocal increase of H3K27ac levels within the *Epha2* gene promotor leads to an increased expression of EPHA2 that stimulates DNA damage induced pro-survival signaling via the AKT kinase pathway resulting in radiation resistance.

Based on these findings we dissected the molecular mechanism of AKT hyperactivation in H3K27me3-deficient medulloblastoma. We found that an epigenetic switch from transcriptionally repressive H3K27 tri-methylation to transcriptionally activating H3K27 acetylation occurs in H3K27me3-deficient cells [18]. Interestingly, a ChIP-Seq analysis revealed that this switch occurs at specific genomic loci altering the transcriptional profile [18]. Among the genes that were upregulated due to the epigenetic switch was EPHA2, a tyrosine kinase that can stimulate the AKT signaling pathway. Using multiple experimental techniques we have established that loss of H3K27me3 induces radiation resistance in medulloblastoma via the pro-survival EPHA2-AKT signaling axis that is activated in response to DNA damage induction [18]. Next, we sought an approach to target radiation resistance in H3K27me3-deficient medulloblastoma. Since bromodomain proteins help facilitating the CBP/p300-dependent H3K27 acetylation [19] that is responsible for EPHA2 upregulation we tested the ability of small molecule BET inhibitors to mitigate radioresistance in H3K27me3-deficient medulloblastoma cells. In fact, BET inhibition was highly effective in restoring radiation response by suppressing H3K27ac levels, inhibiting EPHA2 overexpression and blocking excessive AKT signaling [18]. Together, our study has set a foundation for an epigenetically guided approach to diagnosis and treatment of medulloblastoma (Figure 2).

In view of our recent findings highlighting the role of the histone mark H3K27me3 in radiation response of medulloblastoma the question arises whether H3K27me3 deficiency is responsible for a poor response to radiotherapy in PFA ependymoma and DMG leading to dismal clinical outcomes of these tumors. Importantly, similar to medulloblastoma a global increase in H3K27 acetylation levels has been observed in H3K27me3-deficient PFA ependymoma and DMG [13, 20, 21]. However, the role of this epigenetic switch in regulating radiation response in these brain tumors remains unknown. Based on our findings in medullobastoma we hypothesize that loss of H3K27me3 may be responsible for radiation resistance in PFA ependymoma and DMG resulting in frequent recurrences and disease progression after radiotherapy. Interestingly, multiple studies have demonstrated radiation resistance and worse clinical outcomes associated with low H3K27me3 levels in additional cancer types including esophageal cancer [22] and colorectal cancer [23]. These data indicate a universal role of this epigenetic mark in radiotherapy response that is not limited to medulloblastoma.

Together, these lines of evidence suggest that combining BET inhibition with radiotherapy could be an effective strategy to improve treatment response and survival in H3K27me3-deficient medulloblastoma as well as DMG and PFA ependymoma patients. BET inhibitors have been proposed as therapeutic agents for H3K27M-mutant DMG [24] and PFA ependymoma [25] based on their ability to attenuate tumor cell growth *in vitro* and *in vivo*. However, these agents have been primarily tested as monotherapy in these tumor models. Instead, we propose a concurrent use of BET inhibitors with radiation in pediatric patients undergoing treatment for H3K27me3-deficient brain tumors. Following preclinical testing, this treatment approach can be translated into clinic in a timely manner introducing a significant improvement of the standard-of-care radiotherapy for newly diagnosed patients. In summary, our discovery of H3K27me3 as an epigenetic marker of radiation resistance in medulloblastoma has a potential to open new therapeutic avenues for additional types of pediatric brain tumors including DMG and PFA ependymoma.
